# Understanding bio‐based polymers: A study of origins, properties, biodegradation and their impact on health and the environment

**DOI:** 10.1002/2211-5463.70183

**Published:** 2025-12-12

**Authors:** Sabina Kolbl Repinc, Blaž Stres, Mirica Karlovits, Igor Karlovits, Petra Jerič, Ondrej Panák, Anja Verbič, Blaž Likozar, Uroš Novak

**Affiliations:** ^1^ Department of Catalysis and Chemical Reaction Engineering, National Institute of Chemistry Ljubljana Slovenia; ^2^ Faculty of Civil and Geodetic Engineering University of Ljubljana Slovenia; ^3^ Jožef Stefan International Postgraduate School Ljubljana Slovenia; ^4^ Faculty of Mechanical Engineering University of Maribor Slovenia

**Keywords:** Bio‐based polymers, Biopolymers, Degradation, Environmental impact, Functional material properties, Safe and Sustainable by Design

## Abstract

A growing demand for sustainable materials across various industries has sparked an increasing interest in bio‐based polymers as eco‐friendly alternatives to conventional fossil‐based polymers. Sourced from renewable materials, bio‐based polymers offer significant advantages, such as biocompatibility, the ability to modify their functional properties for specific applications and, increasingly sought after, the capability for biodegradation. This review article provides an overview of bio‐based polymer sources, discussing their unique functional properties, environmental impact and potential for end‐of‐life options, such as composting and anaerobic digestion. It highlights the importance of ensuring human health and environmental hazard assessment, by incorporating principles like a Safe and Sustainable by Design (SSbD) approach and assessing the product's life cycle (LCA). The dual role of the anaerobic digestion of biodegradable polymers and its potential for methane generation is reviewed, emphasising its contribution to reducing environmental impact and renewable energy production through waste management. Lastly, possibilities of applications in different industries and future market trends are reviewed. By integrating current knowledge, this review highlights the potential of bio‐based polymers in advancing sustainability across various sectors, while addressing key existing challenges and future opportunities in their development, production, and application across various sectors, while addressing key existing challenges and future opportunities in their development, production and application.

## Graphical abstract

This review provides an overview of bio‐based polymer sources, their unique functional properties and their environmental impact, and addresses their role as sustainable alternatives. It discusses end‐of‐life options, including composting and anaerobic digestion for renewable energy. The article highlights the importance of Safe and Sustainable by Design and Life Cycle Assessment, as guiding frameworks in research, development and real‐world implementation.

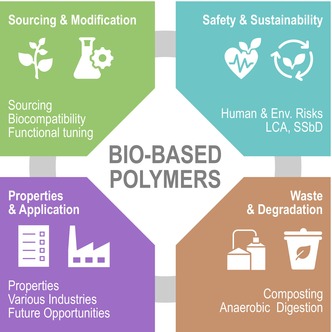

**Abbreviations**
AD: anaerobic digestion
BMP: biochemical methane potential
CA: cellulose acetate/cellulose diacetate
CMC: carboxymethyl cellulose
CNF: cellulose nanofibers
CO_2_
: carbon dioxide
CO_2_‐eq: carbon dioxide equivalent
DNA: deoxyribonucleic acid
EoL: end‐of‐life
HRT: hydraulic retention time
LCA: life cycle assessment
PBAT: polybutylene adipate terephthalate
PBS: polybutylene succinate
PBSA: poly(butylene succinate‐co‐adipate)
PCL: polycaprolactone
PE: polyethylene
PET: polyethylene terephthalate
PGA: polyglycolic acid
PHA: polyhydroxyalkanoates
PHB: polyhydroxybutyrate
PHBV: polyhydroxybutyrate‐co‐valerate
PLA: polylactic acid
PVA: polyvinyl alcohol
SSbD: safe and sustainable by design
TPS: thermoplastic starch
VS: volatile solids

We appear to be on the path toward a paradigm shift in materials science, with a growing emphasis on developing and adopting plastics made of bio‐based and biodegradable polymers, driven by increasing environmental concerns and the urgent need for sustainable alternatives to traditional polymers. Traditional polymers, primarily derived from fossil fuels, are associated with persistent pollution, greenhouse gas emissions and ecological degradation [1]. Despite widespread use, reliable and eco‐friendly end‐of‐life options are limited, often leading to long‐term pollution and landfill accumulation. This highlights the critical need to design bio‐based and biodegradable polymers capable of complete natural degradation. Biodegradable polymers produced from renewable biomass are gaining attention due to their potential to reduce ecological footprints and promote circular economy models across packaging, agriculture and biomedical sectors [2]. Prominent examples such as polylactic acid (PLA), polyhydroxyalkanoates (PHA) and cellulose‐based materials offer promising pathways to replace fossil‐based polymers. However, scalability, cost and performance challenges remain, mainly due to the lack of reliable, scalable alternatives with comparable functionality to fossil‐based plastics [3, 4].

The choice of terminology presents an initial challenge. While the International Union of Pure and Applied Chemistry actively discourages the use of the term ‘bioplastic’ [5], it is still widely used within both the industrial and scientific literature. European Bioplastics [6] sees ‘bioplastics’ as an extensive group of materials that are either ‘bio‐based’, or ‘biodegradable’, or carry both properties. The classification shown in Fig. 1 is based on: (i) feedstock origin (bio‐ or fossil‐based) and (ii) biodegradability, forming four quadrants: bio‐based and biodegradable (e.g., PLA, PHA), bio‐based but nonbiodegradable (e.g., bio‐PE, bio‐PP), fossil‐based but biodegradable (e.g., polybutylene adipate terephthalate (PBAT)), and fossil‐based nonbiodegradable (e.g., PET, PE).

**Fig. 1:**
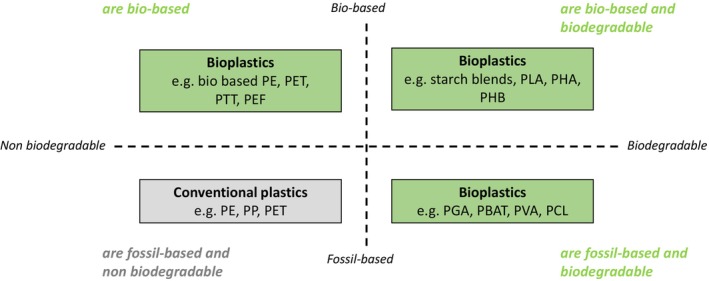
Classification of plastics based on feedstock origin and/or their biodegradability. Adapted from [6].

The use of more descriptive terminology is encouraged to avoid the term ‘bioplastics’ [5, 7] The present review article follows the terminology that refers to:

- ‘bio‐based’ polymers, as those entirely or partly derived from living matter (biomass) [5, 6], with a subgroup of ‘biopolymers’, which are entirely formed by living organisms [5];
- ‘biodegradable’ polymers, as those that degrade, when biological activity lowers the molar masses of their component macromolecules, and break down into natural by‐products like water, carbon dioxide and compost [5, 6].

The interchangeability of the terms ‘polymer’ and ‘plastic’ can be attributed to the understanding of ‘plastic’ as a polymeric material that may contain certain additives to improve its properties [5]. This interchange is inevitable across various sectors. However, the descriptive terminology for bio‐based and biodegradable plastics/polymers (while avoiding the term ‘bioplastics’) has been achieved and formally adopted by the European Union (EU) [8, 9].

This review provides a concise discussion of the origins, physical, mechanical and functional properties of bio‐based polymers, their market potential, Safe and Sustainable by Design (SSbD) methodologies, and the role of aerobic digestion as a valuable end‐of‐life (EoL) option for bio‐based polymers.

## Life cycle assessment

The significance of sustainability in material science extends beyond environmental benefits to include social and economic dimensions such as resource efficiency, waste reduction and lifecycle management [10]. Furthermore, conventional plastic production has a considerable environmental footprint, characterised by significant CO_2_ emissions, high water usage and substantial energy consumption. To ensure sustainability targets are met, the environmental impact of bio‐based and biodegradable polymers must be assessed throughout their entire life cycle. Life cycle assessment (LCA) is a tool that can be used to measure the inputs (e.g., energy, resources) and outputs (e.g., emissions, waste) of a product's lifecycle, converting these into impact categories such as climate change, resource depletion and ecotoxicity [11]. It provides a comprehensive perspective on environmental sustainability and is ISO‐standardised [12]. Incorporating LCA during the innovation process facilitates the quantification of the impact of design objectives and enables the identification of hotspots for improvement [2]. Since bio‐based polymer data can be incomplete [13], clearly defining the LCA's scope prevents misinterpretation. The typical process involves the following stages [14]: Firstly, an inventory analysis (comprising the compilation of all inputs/outputs, including raw materials and emissions); secondly, an assessment of chemical emissions (types and quantities of chemicals used/emitted, including toxic substances); thirdly, a characterisation of factors (using models like USEtox [15] to evaluate toxicity impacts); fourthly, an impact assessment (identifying stages with significant toxicological risks); and fifthly, hotspot identification (pinpointing critical manufacturing or waste stages for mitigation). Strategies such as safer chemical substitution, improved waste treatment and process optimisation have been shown to reduce risks, with LCA supporting continuous improvement and chemical safety integration [1, 10].

## Safe and sustainable by design

**Fig. 2:**
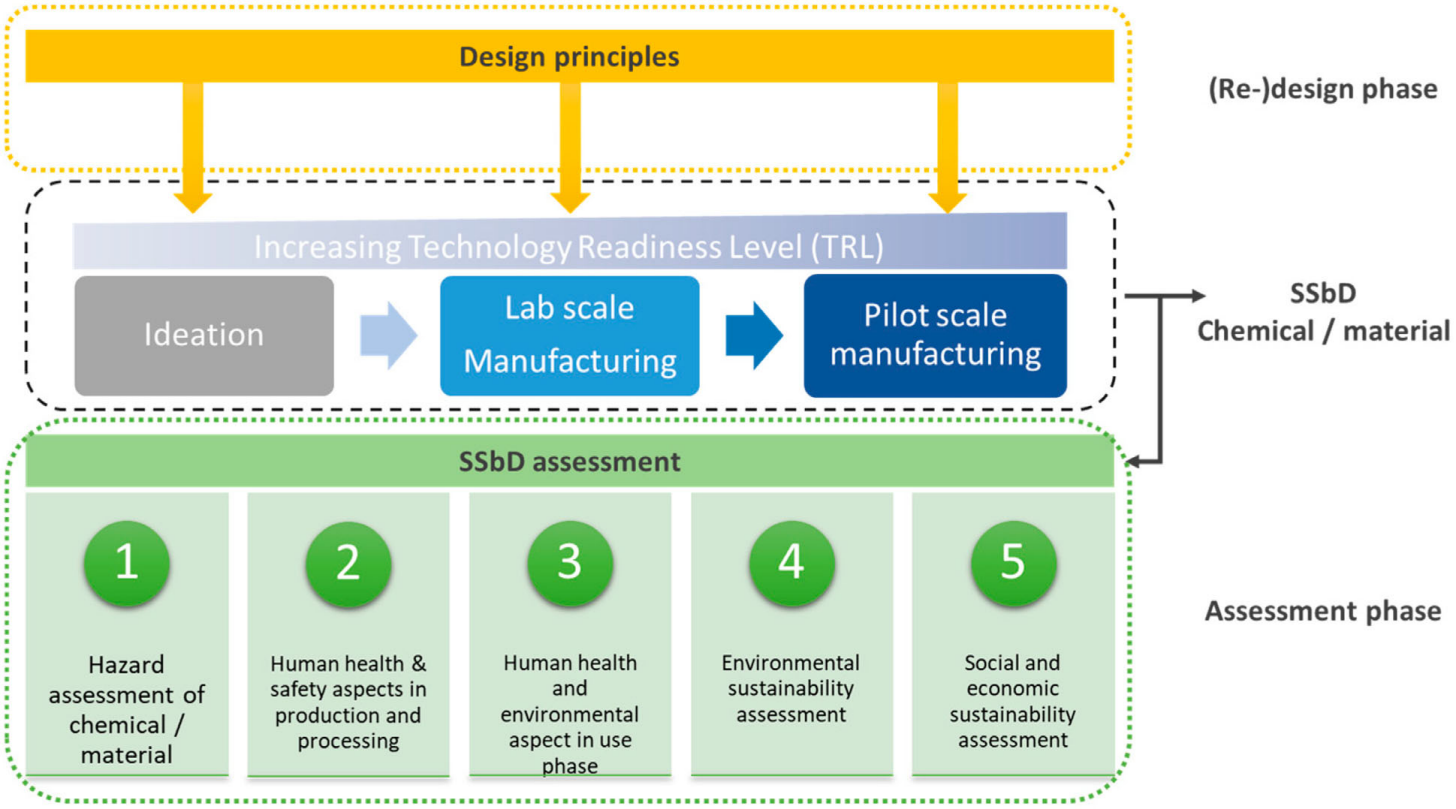
Schematic representation of Safe and Sustainable by Design framework. Reprinted with permission from [18].

SSbD is a framework, developed by the European Commission for the purpose of minimising the health, climate and environmental impact of chemicals, materials, technologies and products throughout their lifecycle [16, 17]. Certain approaches consider both societal and economic value [2, 18]. SSbD is applicable to both new and existing products through an iterative process involving: (a) (re)design with guiding principles to guide the development process and (b) assessment of safety and sustainability [19, 20, 21]. The assessment is comprised of five distinct steps, as presented in Fig. 2: (1) hazard assessment, (2) health and safety in production, (3) impacts during use, (4) environmental sustainability and (5) optional socio‐economic evaluation [2].

## Methods

This literature review provides a critical synthesis of the literature on the properties, degradation and impacts of bio‐based polymers on human health and the environment. The objective of the search was to identify scholarly publications that define the current understanding of the topic. A targeted search was conducted across databases such as Scopus, Web of Science, Google Scholar and Zenodo. The selection criteria were based on thematic relevance, a clear focus on bio‐based polymers and the quality and clarity of the experimental and theoretical evidence presented, as well as the conclusions drawn. The selection was intended to facilitate a critical discussion, highlighting ongoing debates and emergent research rather than achieving exhaustive coverage, with a preference for the most recent publications (2025–2020).

## Origin and properties of bio‐based polymers

### Origin of bio‐based polymers

Bio‐based polymers originate from diverse natural sources, such as plants, animals and microorganisms, and are obtained through direct extraction or microbial synthesis [8]. Polysaccharides like starch, derived from crops such as corn, potatoes, bananas, tapioca, wheat, rice, yam, sago and buckwheat, are primary renewable resources. Cellulose, mainly from softwood, and pectin from citrus fruits are also major sources. Chitin, abundant in crustacean shells, and seaweed polysaccharides, such as alginate, agar and carrageenan, further expand the selection of bio‐based polymers [22, 23]. Proteins—including casein, whey, gelatin, zein and animal proteins—are also used as bio‐based polymers [23, 24, 25]. Lignin, sourced from lignocellulosic biomass and agricultural waste, is a key bio‐based polymer by‐product [26, 27]. Microbial bio‐based polymers like polyesters, PHAs, and PLAs are produced by bacteria under various conditions, often as lipid granules [22]. Microalgae such as spirulina and chlorella, rich in proteins and carbohydrates, are promising alternatives. Some bio‐based polymers, like bio‐polypropylene (bio‐PP) and bio‐polyethylene (bio‐PE), are synthesised from bio‐based monomers derived from renewable crops like corn and sugarcane, using chemical processes to produce polymers structurally similar to conventional types. Although bio‐based, these materials are often nonbiodegradable because their chemical structure resists microbial breakdown, similar to fossil‐based polymers.

### Overview of the physical, mechanical and functional properties of bio‐based polymers

**Fig. 3:**
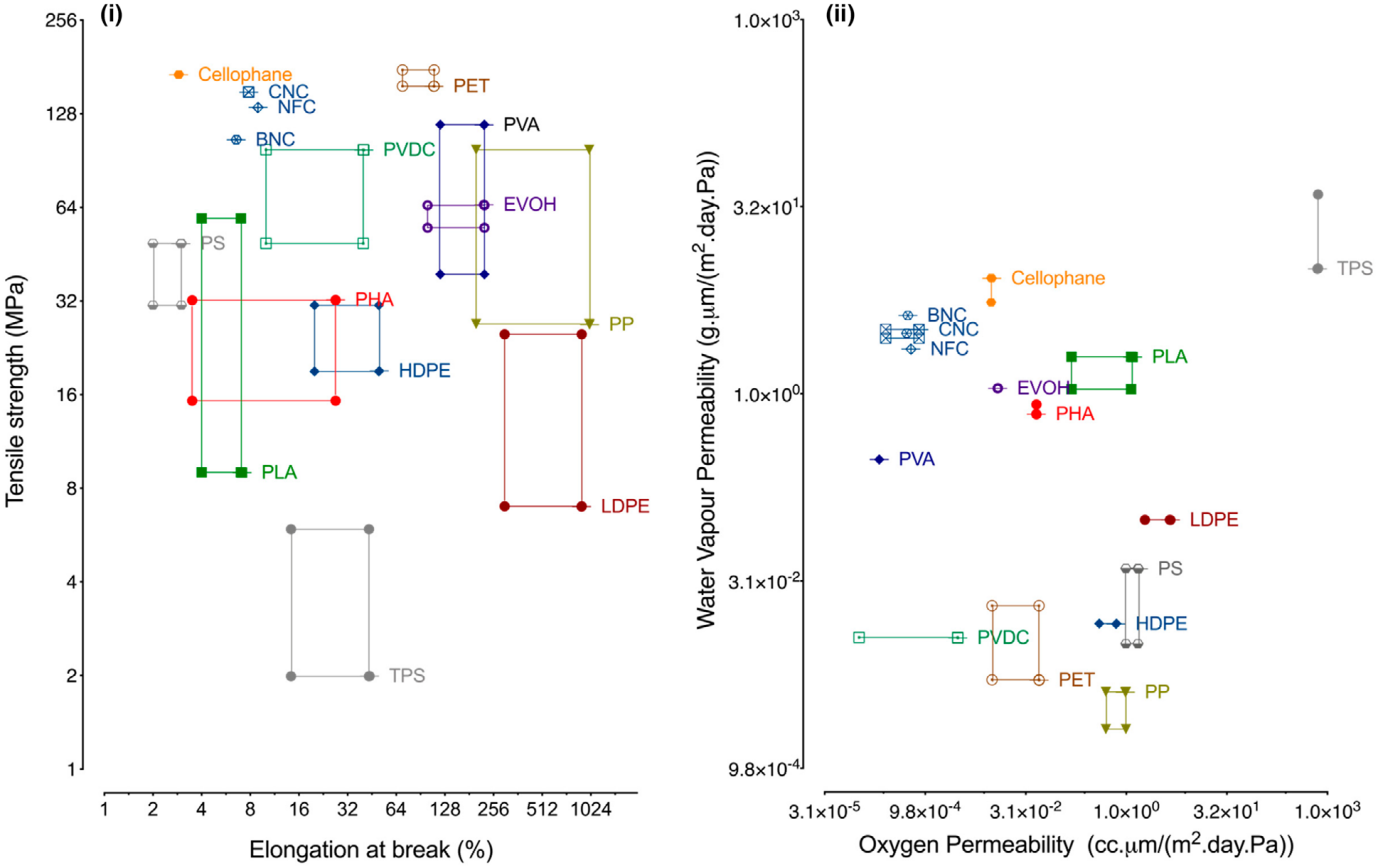
Tensile strength and water vapour permeability properties of synthetic and bio‐based polymers. Reprinted with permission from [32].

Understanding the physical and mechanical properties of bio‐based polymers, such as tensile strength and elasticity, is essential for their practical application across various industries. Tensile strength is defined as the maximum stress a material can withstand before failure, whereas elasticity reflects its ability to return to its original shape after deformation. These properties vary significantly depending on the polymer type, reinforcements, processing conditions and additives employed [28, 29]. For instance, PLA exhibits high tensile strength and elastic modulus (2500–3500 MPa), suitable for durability‐focused applications, whereas starch‐based plastics exhibit lower values (~10–100 MPa). Reinforcing bioplastics with natural fibres has been shown to further enhance their performance, while modifications such as the incorporation of hydroxyvalerate units improve the flexibility of copolymers such as PHBV [30, 31]. In order to make informed material choices, it is crucial to evaluate these properties using standardised test methods such as ASTM D638, ISO 527, ASTM D882, ISO 178 and ASTM D790. These standardised tests enable the comparison of bio‐based polymers with conventional fossil‐based polymers, ensuring their suitability for specific applications. Figure 3 provides a visual comparison [32]. Most bio‐based polymers are not as ductile as their fossil‐based counterparts, but they can withstand comparable tensile forces. Bio‐based polymers usually underperform fossil‐based ones in water vapour permeability.

**Fig. 4:**
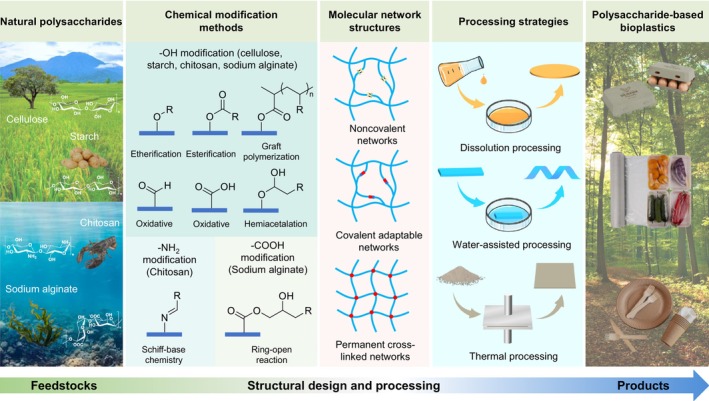
Examples of sources of biopolymers and their advancement via chemical modifications toward improved properties and polysaccharides applications. Reprinted with permission from [33].

Bio‐based polymers like cellulose, starch, carrageenan, pectin, pullulan, soy and whey proteins, PLA, polyvinyl alcohol (PVA), PHAs, and polyhydroxybutyrate (PHB) serve as barriers against gases and moisture, vital in food preservation [25]. Various natural biopolymers can be chemically modified to enhance their physicochemical properties and broaden their range of functional applications (Fig. 4). Advances—such as composites, multilayer films or adding nanoparticles—have improved their barrier and functional properties, sometimes exceeding polyethylene terephthalate (PET's) performance [34, 35]. Besides barrier functions, biopolymers can provide antimicrobial activity, either inherently or via engineering. For example, chitosan disrupts bacterial cell walls, while egg proteins contain lysozyme and ovotransferrin [36, 37]. These properties can be enhanced by incorporating inorganic compounds, extracts or synthetic antimicrobials, providing resistance against bacteria (e.g., *S. aureus*, *E. coli*) and fungi [25].

## Improving properties of bio‐based polymers: Blending techniques and scale up for diverse applications

### Blending, extrusion and scale up of bio‐based polymers

One of the most effective strategies for enhancing the properties and functionality of bio‐based polymers is through the process of blending. By combining different bio‐based polymers, complementary characteristics can be achieved, allowing the merging of their desirable properties while mitigating their weaknesses. Brittleness, low mechanical properties and poor thermal resistance are limitations of many bio‐based polymers, which can be overcome by polymer blending, which physically or chemically combines polymers with complementary properties. In a review paper by Fredi and Dorigado [38], the authors made a distinction between nonreactive techniques. It was posited that the use of block or graft copolymers can increase compatibility, though property enhancements are often modest improvements of the properties of a bio‐based polymer. Research has found that what is more impactful are reactive methods, which functionalise and form compatible copolymers *in situ* during the melt‐blending process. The incorporation of nanoparticles has been demonstrated to be an effective method of achieving compatibility through the processes of interface localisation and morphology control. The addition of compatibilisers, such as maleic anhydride, dicumyl peroxide and Joncryl, to enhance the blending process is a common practice. These compatibilisers are utilised to address various challenges, including poor interfacial adhesion between components, limited scalability and variability in biodegradation rates. Another notable trend in the field of blending is the integration of macro and nanoparticles from bio‐based agricultural by‐products. Examples include coffee grounds [39, 40], wood dust [41], waste paper [42], different stalk types and cellulose‐based sourcing like nanocellulose and lignin [35, 43, 44].

Extrusion remains the prevailing technique for shaping bio‐based polymers. Nonetheless, its implementation in the industrial environment still faces several challenges that limit large‐scale and high‐performance applications. During the process, thermal and shear energies are applied to the material, resulting in thermal sensitivity and biopolymer stability [45, 46], or in repeated regrinding and extrusion for potential recyclability purposes [47]. It has been demonstrated that bio‐based polymers, like PLA and PHB, are more thermally sensitive than petroleum‐based plastics. Their narrow processing windows and lower degradation thresholds require careful extrusion control, often with the aid of stabilisers, plasticisers or blending strategies. In contrast, PE and PP exhibit robust thermal stability, facilitating easier processing and enhanced tolerance to industrial conditions. The differences are presented in Table 1.

**Table 1:** Thermal and processing properties of polymers relevant to blending and extrusion.

Property	PLA	PHB	PEP	PPT
Onset (°C)	280–300	260–280	350–400	320–350
Tmax (°C)	330–370	290–310	450–500	400–450
Processing temp. (°C)	180–210	160–180	180–250	200–250
Thermal stability	Moderate	Low	High	High
Degradation risk	High (hydrolysis)	Very high	Low	Low
Drying required	Yes	Yes	No	No

### Current uses and future potential in various industries

Bio‐based and biodegradable polymers have been increasingly recognised and explored as sustainable alternatives to conventional materials in numerous industrial sectors, offering renewability, biodegradability and reduced environmental impact. Although bio‐based and biodegradable polymers currently account for only 0.5% of the approximately 414 million tonnes of polymers produced annually, the market is expanding rapidly. Production is projected to rise from 2.1 million tonnes in 2023 to 5.7 million tonnes by 2029 [48]. Packaging remains the leading application of bio‐based and biodegradable polymers, accounting for about 45% of total production (1.12 million tonnes in 2024), including films and bottles [48]. However, emerging applications in the domains of automotive, electronics, textiles and soil‐degradable agricultural films are also projected to expand [49]. In the field of healthcare, biopolymers such as chitosan and PLA have a wide range of applications. This includes wound dressing, tissue engineering and drug delivery systems, where antimicrobial and barrier properties can enhance infection control and controlled release of therapeutics [36, 50]. Biopolymer membranes and flocculants are also being applied in water treatment and environmental remediation, due to their ability to filter contaminants while remaining eco‐friendly [51]. In the field of textiles, coating from bio‐based polymers has been employed to improve water resistance and provide antimicrobial protection, while in the automotive and electronic industry, lightweight and moisture‐resistant components enhance both performance and sustainability [52, 53, 54]. In the agricultural sector, biodegradable films and seed coatings provide protection against moisture loss and microbial contamination, while naturally degrading in soil [55]. Emerging innovations, such as high‐barrier and multifunctional biopolymers, enabled by incorporating nanomaterials, natural extracts and chemical modifications, further improve durability, stability and applicability under demanding conditions [49]. The ongoing pressure from regulatory bodies, coupled with the growing consumer demand for sustainable and health‐conscious materials, is a key driver of innovation.

**Fig. 5:**
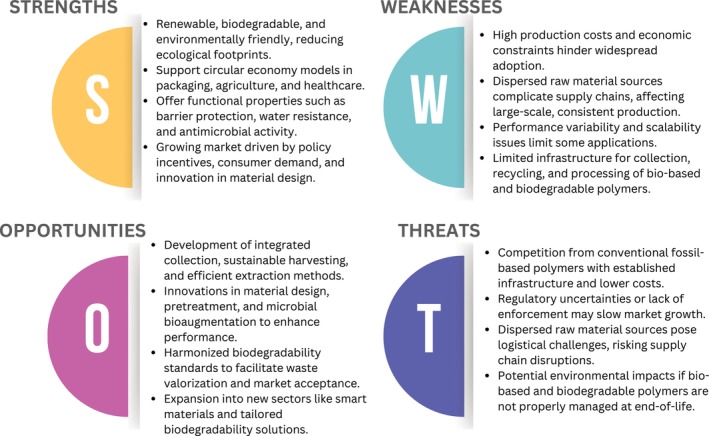
SWOT analysis of bio‐based and biodegradable polymers.

Despite rapid development, challenges remain in terms of scaling production and managing waste. Future research will be primarily driven by bio‐based composites, innovative materials and tailored biodegradability solutions, thereby paving the way for a sustainable future and addressing issues of resource depletion and climate change [56]. Furthermore, in order to unlock their full potential while minimising environmental impact, there is a necessity for the development of integrated collection, sustainable harvesting, efficient and greener extraction techniques [57]. Figure 5 provides an illustrative representation of the aforementioned aspects in the form of a strengths, weaknesses, opportunities and threats (SWOT) analysis.

## Impact on human health and environment

Bio‐based polymers, such as chitosan, gelatin, carboxymethyl cellulose (CMC), cellulose, PVA, PLA, gellan gum and dextran, are widely used in bioengineering [50]. However, the presence of the term bio‐based or biodegradable does not necessarily guarantee low toxicity. Zimmermann *et al*. [58] found that the toxicity of bio‐based products, especially starch and cellulose‐based, was comparable to that of fossil‐based plastics, as studied on 43 commercially available consumer products. Lestido‐Cardama *et al*. [59] observed that novel bio‐based monomers and oligomers frequently exhibit an absence of toxicity data. *In silico* toxicity prediction has the potential to address data gaps; however, it should be noted that the accuracy declines as molecular weight increases [60]. Liu *et al*. [61] discovered that starch‐based microplastics accumulate in mouse organs, resulting in microstructural damage and metabolic disturbances. Shi *et al*. [62] suggest that poly(lactic‐co‐glycolic acid) nanoparticles used in medical applications may contribute to cardiovascular stenosis. It is important to note that nanocellulose also raises toxicity concerns. As posited by Yanamala *et al*. [63], oxidative stress and lung inflammation have been reported as a consequence of exposure to cellulose nanocrystals, while Yamashita *et al*. [64] found that cellulose nanofibrils induced macrophage buildup, an increase in lung weight and inflammation.

The environmental fate of bio‐based polymers is another critical concern. PHB microbeads have been demonstrated to cause harm to fish tissues [65], while xylan‐starch blends have been shown to be nonecotoxic [66]. Furthermore, it has been demonstrated that biodegradation processes have the capacity to induce alterations in microbial ecosystems. De Bernardi *et al*. [67] found that composted bio‐based polymers did not affect earthworm fertility. However, they did induce DNA damage at high concentrations and impact fungal communities. Boisseaux *et al*. [68] observed low toxicity from pork gelatine, fish gelatine, chitosan and macroalgae‐based biopolymers in Daphnia magna, though water acidification was a concern. As Koumentakou *et al*. [69] summarise, the use of chitosan and its derivatives is generally low‐risk, except for certain marine species, although nano‐chitosan may increase toxicity. PLA‐based plastics can harm marine cells [70]. Cucina [71] emphasised that truly biodegradable bio‐based polymers pose a lower environmental risk than their fossil‐based counterparts.

### LCA of bio‐based polymers

Plastics derived from bio‐based polymers offer a promising alternative, with LCA indicating that the production of 1 kg of PET results in 2.15–2.50 kg carbon dioxide equivalent (CO_2_‐eq), whereas the production of bio‐based plastics such as PLA and PHA emits substantially lower quantities, ranging from 0.50 to 0.80 kg CO_2_‐eq. Starch‐based films have been shown to emit only 0.20–0.50 kg CO_2_‐eq, with lower energy demands than PET [72]. LCA reports on bio‐based polymers show some clear environmental trade‐offs [73, 74, 75, 76, 77]. Bio‐based materials excel at reducing fossil fuel depletion and sequestering carbon. However, environmental burdens are associated with feedstock sourcing. Worse performance is reported for categories such as agricultural land occupation, water use and eutrophication potential. Collection and EoL management are critical for amplifying their climate benefits [76, 78, 79].

Recent research highlights the environmental benefits of using nonedible feedstocks for bio‐based polymer production. Specifically, producing PLA from food waste significantly reduces its impacts on climate change, eutrophication and ecotoxicity compared to using corn [80]. Furthermore, PHB from lignocellulosic biomass has a lower environmental impact than PLA from the same source [1]. Similarly, poly‐dihydroxybutyric acid made from nonedible carbon feedstock performs better than PLA across most environmental categories [81]. The environmental performance of PBAT produced from second‐generation bio‐based feedstocks improves by up to 93% compared to its fossil‐based counterpart [82], and the utilisation of biomass by‐products further reduces the overall environmental impact of bio‐based polymers [83]. However, it is important to note that a poorly sourced bio‐based polymer could still have a significantly worse climate impact than a petrochemical alternative [84]. Nano‐sized bio‐based additives from chitin, starch or cellulose, that provide improvement of polymer properties, carry a negative environmental impact due to high chemical and energy consumption in the production phase. Chemical emissions from the growth medium and electricity consumption have a negative environmental impact in the case of bio‐based polymer produced from *Spirulina* microalgae [85].

The preferred EoL scenario suggestions vary by polymer and approach, which consider idealised or real EoL options. In the study by Newby *et al*. [86], anaerobic digestion resulted in the lowest net environmental impacts across most categories due to the environmental offsets from energy generation. On the other hand, mechanical recycling of the PLA rug was the best‐performing scenario in a cradle‐to‐grave case study over 100 years [87], because it retains the biogenic carbon captured by the source material (sugar beets). Incineration with energy recovery was reported [88] to be favourable for PLA derived from corn stover. Industrial composting was found to be the least favourable EoL option for short‐life PHBV‐based products, favouring mechanical recycling [89]. Schwarz *et al*. [90] demonstrated a novel depolymerisation technology as an effective chemical recycling method for PLA. Sustainability outcomes for PLA and PHA bio‐based polymers were reported to be highly dependent on regional factors such as energy grid mix and industrial composting infrastructure, rather than the material itself [91]. Transportation distances can highly influence the impact [86].

Scaling up bio‐based polymer production [89, 92], purification [88], solvent recovery [93] and replacing the standard electricity source with solar energy [85] can provide a significant reduction in environmental impacts. It was also reported that proper accounting of biogenic carbon in PLA assessment can reduce GWP of about 79% [94].

### SSbD approaches in the development of bio‐based polymers

While literature frequently promotes bio‐based polymers using broad labels such as ‘safety’ (referring to biocompatibility or intrinsic nontoxicity) and ‘sustainability’ (due to renewable sourcing and biodegradability) [3, 95, 96, 97, 98], such assessments are often either assumed or based on selected factors rather than a holistic approach. Recent studies on SSbD frameworks have revealed that only a limited number of sources have adopted this approach for particular bio‐based polymers. Caldeira *et al*. [99] found that only one of their 155 reviewed sources (mostly conceptual) included bio‐based and biodegradable polymers. Elser and Buchmeiser's [100] study concluded that SSbD is the optimal assessment method for fibre‐reinforced bio‐based polymer composites. However, the study did not provide concrete examples to support this assertion. Marques *et al*. [101] also propose a design for promoting safe and sustainable biorefineries, driven solely by conceptual considerations.

Zhen and Chew [102] used a multi‐criteria optimisation framework to evaluate six different process routes for the production of PHA from nonfood crops. The selection of the most suitable road for PHA production was made possible by assessing economic factors and sustainability. The safety factors were not considered during the optimisation process. Todea *et al*. [103] demonstrated an integrated experimental and computational approach for rapid screening of bio‐based polyester oligomers, with a particular focus on their marine biodegradability and ecotoxicity. Oliaei *et al*. [104] claimed to follow the SSbD principles, but without closed disclosure about safety and sustainability metrics taken into consideration. Nonetheless, certain safety and sustainability principles can be identified in the context of material sourcing, molecular design that allows chemical recycling to monomer without destroying the fibre, recycling conditions avoiding strong acidity or high temperatures and process safety through using a catalyst approved for biomedical applications. Zappatera *et al*. [105] designed and synthesised bio‐based functionalised oligoesters by pairing predictive modelling with green, low‐input synthetic routes. Despite the absence of disclosure regarding SSbD workflow metrics, the approach employed *in silico* prescreening to rationally select promising polymer structures, focusing on fully bio‐based sourcing and predicting performance‐relevant properties. The synthesis minimised environmental impact, and the final oligoester was assessed for biodegradability and ecotoxicity.

Medina *et al*. [106] have demonstrated a SSbD screening tool in a case study on organic aerogel composites, based on a combination of raspberry fruit pulp waste, nanocellulose and carbon nanotubes. The starting materials and the final aerogel form and installation have an influence on safety and environmental exposure, while synthesis processes, primarily due to electricity use for drying, dominate the sustainability impact, which could be optimised by recycling solvents and catalysts and realising energy savings during the product's use phase. Smith *et al*. [107] conducted a comprehensive SSbD evaluation of alginate extraction from *Macrocystis pyrifera*. A chemical hazard assessment was performed on 23 existing alginate extraction methods by evaluating the hazard levels of their solvents and reagents to human and environmental health. A partial life cycle assessment was then performed on three protocols utilising low‐to‐moderate hazard chemicals. A clear trade‐off between the environmental impact and the physicochemical properties of the resulting alginate for high‐performance bio‐based polymer products has been highlighted.

The development of integrated safety and sustainability assessment methods is imperative to facilitate their practical use. The holistic SSbD approach has been applied in a variety of case studies utilising bio‐based polymers [108, 109] and the detailed reports can be expected in the near future.

## Degradation of bio‐based polymers

### Environmental degradation

The assessment of biodegradation and disintegration helps understand plastic waste in different environments. The degradation of polymers is a process that requires specific conditions to occur. In case of mismanagement, there is a potential for environmental harm to be caused [110]. PLA, one of the most common bio‐based polymers, disintegrates into microplastics faster than fossil‐based plastics, posing threats to biota, especially since it may not degrade naturally without enzymes, as confirmed by several studies [111, 112, 113, 114]. Various microorganisms can degrade polymers: about 53 species affect bio‐based plastics, while only 16 species have been shown to degrade fossil‐based plastics. Microorganisms like *Aspergillus, Bacillus, Fusarium, Penicillium* and *Streptomyces* can degrade both types, with *Amycolatopsis* and *Cladosporium* capable of degrading PLA, polycaprolactone (PCL) and polybutylene succinate (PBS) [115]. The external environment that supports or prohibits microbial community, the intrinsic physicochemical properties of the polymer and the characteristics of any additives largely influence the biodegradation in real‐life natural conditions [116, 117, 118]. Therefore, the biodegradation behaviour of bioplastics in various environments needs to be thoroughly explored, together with ecotoxicity. Moreover, an appropriate assessment of real‐life EoL options is crucial to facilitate their sustainability potential [77].

### Anaerobic degradation of bio‐based polymers for methane production

Bio‐based polymers represent a sustainable alternative to conventional fossil‐derived plastics, as they are produced from renewable biological resources and, in many cases, are also biodegradable. Their end‐of‐life management can be accomplished through biological processes such as composting or anaerobic digestion (AD).

AD provides a sustainable and energy‐efficient method for managing bio‐based polymer waste, enabling the transformation of biodegradable polymers into methane‐rich biogas and nutrient‐dense digestate through microbial activity under oxygen‐free conditions [119, 120]. The efficiency of the anaerobic degradation of bio‐based polymers is influenced by environmental conditions, particularly temperature, as well as the specific properties of the bio‐based polymer in question. Under mesophilic conditions (30–38 °C), four out of ten tested bio‐based polymers showed biodegradation levels ranging from 57.9% to 84.6%, largely facilitated by bacterial orders such as *Anaerolineales, Bacteroidales* and *Clostridiales* [120]. In contrast, thermophilic conditions (49–57 °C) support faster and more complete degradation, with five bio‐based polymers achieving up to 95.7% biodegradability through the activity of thermophilic bacteria such as *Coprothermobacter* and methanogenic archaea such as *Methanothermobacte*r [120]. PHB has demonstrated high anaerobic biodegradability, with methane conversion efficiencies between 80% and 98% under thermophilic conditions, especially when codigested with organic waste [119]. PLA, however, is more recalcitrant and typically requires alkaline or thermal pretreatment to achieve comparable biodegradation (~70%). Various pretreatment strategies have been developed to enhance the degradability of resistant polymers like PLA and PBS. Alkaline and thermal treatments significantly improve degradation by increasing surface accessibility and reducing crystallinity. Thermo‐alkaline pretreatment, such as exposing PHB to 70 °C in NaOH, achieved over 70% abiotic degradation in a few hours [121]. Codigestion with organic‐rich substrates such as sewage sludge improves microbial diversity and process stability, thereby further enhancing methane production [122].

Bio‐based polymers vary significantly in AD behaviour, influenced by their structure (e.g., crystallinity and aromatic content) and process conditions (temperature, hydraulic retention time (HRT), microbes, pH and inhibitors). PHAs (including PHB and polyhydroxybutyrate‐co‐valerate (PHBV)) are fully bio‐based, showing excellent biodegradability under mesophilic and thermophilic conditions. Their aliphatic ester backbone enables rapid microbial conversion, often reaching over 80% of the theoretical methane potential within 30–40 days [120, 123]. Thermoplastic starch (TPS), derived from native starch, degrades quickly due to its hydrophilic, amorphous nature, producing high methane yields within 15–30 days. It is often used to improve the degradability of composite polymeric materials [124]. Fossil‐based PCL degrades under thermophilic conditions within 60 days, facilitated by lipase‐mediated hydrolysis [125]. PLA degrades slowly in mesophilic systems but can yield up to 260 L CH_4_/kg volatile solids (VS) in thermophilic AD after extended retention times (> 80 days) [120]. Its breakdown depends on surpassing its glass transition temperature and microbial activity. PBAT and PBS show limited biodegradability: PBAT achieves only 20–40% degradation thermally, with methane yields below 10%, while PBS yields are around 11–20 L CH_4_/kg VS even under thermophilic conditions [119, 120]. Mixed polymeric materials often degrade unevenly, with biodegradable components such as TPS mineralising faster than recalcitrant fractions such as PLA and PBAT, which can fragment into microplastics. Among bio‐based polymers, PHA and TPS are the most promising for energy recovery due to their high methane yields and compatibility with existing AD systems, especially when codigested with nitrogen‐rich waste [120]. Conversely, PLA, PBAT and PBS require pretreatment or microbial augmentation to improve biogas production. Incorporating additives such as biodegradable fibres or enzymes into plastics offers a future route to enhance *in situ* degradation, though this remains in the early development stages [120]. For a complete overview of the anaerobic degradability and biochemical methane potential (BMP) from bio‐based polymer types and sources, check Table 2.

**Table 2:** Overview of the anaerobic degradability and methane potential from bio‐based and biodegradable polymer types and sources.

Acronym	Full name	Origin	Anaerobic biodegradability	Methane potential (BMP)	Anaerobic biodegradation [%]	Source
PLA	Polylactic acid	Bio‐based	Partial (thermophilic, slow in mesophilic)	4.4–453 L CH_4_/kg VS	4.2–97.5	[120, 121, 125, 126, 127]
PLA blend	Polylactic acid blend	Bio−/Fossil‐based	Moderate (varies by composition)	84–442.6 L CH_4_/kg VS	36.2–80.6	[126]
PHB	Polyhydroxybutyrate	Bio‐based	Excellent	199–518 L CH_4_/kg VS	57.6–93.2	[119, 121, 125, 126]
PHBV	Polyhydroxybutyrate‐co‐valerate	Bio‐based	Excellent	271–397.8 L CH_4_/kg VS	73–96	[126, 128]
TPS	Thermoplastic starch	Bio‐based	High	278.2–338.8 L CH_4_/kg VS	79.6–96.2	[119, 125, 126, 127]
Starch‐based	Starch‐based bioplastics	Bio‐based	High	119–392 L CH_4_/kg VS	72.1–96.4	[125, 126, 127]
PBS	Polybutylene succinate	Fossil‐/Bio‐based	Low to moderate	0.47–11 L CH_4_/kg VS	0.5–4.8	[125, 126, 127]
PBSA	Poly(butylene succinate‐co‐adipate)	Fossil‐/Bio‐based	Moderate (thermophilic preferred)	50.7–164.5 L CH_4_/kg VS	27.9–71.7	[126, 127, 129, 130]
PVA	Polyvinyl alcohol	Fossil‐based	Variable	68.9–71.4 L CH_4_/kg VS	8–29.5	[120, 126, 127]
PET	Polyethylene terephthalate	Fossil‐/Bio‐based	None	Negligible	< 1	[125, 126, 131]
PE	Polyethylene	Fossil‐/Bio‐based	None	Negligible	< 1	[125, 126, 131]
PGA	Polyglycolic acid	Fossil‐/Bio‐based	Limited	Not well established	–	[125, 126]
CA	Cellulose acetate/cellulose diacetate	Bio‐based	Moderate	326.3–519.3 L CH_4_/kg VS (mesophilic)	8.9–100	[126, 127]

### Aerobic degradation (composting) of bio‐based polymers

The composting of bio‐based polymers is an aerobic biological process in which microorganisms such as bacteria and fungi enzymatically break down polymer chains into smaller molecules that are mineralised into CO_2_, H_2_O and biomass [132]. The mechanism of biopolymer composting involves a sequence of enzymatic and microbial processes. Initially, microorganisms secrete extracellular enzymes such as esterases, lipases and cutinases that hydrolyse ester bonds in polymers like polylactic acid (PLA) and polyhydroxyalkanoates (PHA), producing smaller monomers and oligomers [133]. These intermediates are subsequently assimilated and metabolised by microorganisms, resulting in the formation of CO_2_, H_2_O and biomass under aerobic conditions [132]. Industrial composting typically progresses through three thermal stages: a mesophilic phase allowing microbial adaptation, a thermophilic phase (50–70 °C) characterised by intense biodegradation and a final maturation phase [134]. During the maturation phase, temperatures typically decline from the thermophilic peak to mesophilic or ambient levels, allowing microbial communities to stabilise and further break down complex compounds, contributing to the formation of stable humic substances. The rate and extent of degradation of biopolymers in composting are significantly influenced by both the polymer's structural characteristics and environmental conditions. Key polymer factors include: (a) the presence of hydrolysable bonds in the polymer chain, which are susceptible to breakdown via hydrolysis, (b) polymer morphology, where amorphous regions degrade faster than crystalline structures due to easier enzyme access and (c) the molecular weight, with lower molecular weight polymers typically degrading more quickly. Environmental parameters critical to effective biodegradation include temperature—higher temperatures, especially in industrial composting (55–70 °C), accelerate hydrolysis and microbial activity—moisture availability, which facilitates enzymatic reactions, oxygen presence for aerobic microbial degradation, and pH levels that affect microbial populations and enzyme function [135, 136, 137, 138]. The degradation of PLA generally requires industrial composting conditions, characterised by elevated temperatures of around 60 °C, controlled humidity and a composting period of at least 90 days to achieve approximately 90% degradation [136, 138, 139]. In natural settings, such as soil or marine environments, PLA shows much slower degradation and can persist for extended periods. Bio‐PBS is biodegradable under industrial composting conditions.

The extent and rate of its degradation depend on factors such as the polymer's physical form, the composition of the microbial community and the specific environmental conditions within the composting process. Under optimised conditions, biodegradation levels of up to 80% have been reported [140]. On the other hand, Polythioesters (PTE) is resistant to biodegradation and not suitable for composting [141].

## Conclusion

From research and development perspective, bio‐based polymers, including natural biopolymers, are seen as sustainable alternatives to fossil‐based polymers, especially where barrier properties, water resistance and antimicrobial properties are needed. They offer renewability, with some types also offering biodegradability, as well as reduced environmental impact. However, their broader adoption is hindered by economic constraints and market adaptation challenges. These materials, which are derived from various biological sources, support circular economy models across sectors such as packaging, agriculture and healthcare. However, the dispersed raw material sources complicate supply chains, posing logistical and economic challenges for large‐scale, consistent production. Proper assessment of health and environmental impact is necessary to provide clear superiority of bio‐based polymers. Addressing performance consistency, scalability, health and environmental impacts, and cost‐effectiveness remains critical. Innovations in material design, pretreatment, waste collection and microbial bioaugmentation, alongside harmonised biodegradability standards, are vital for advancing the field of bio‐based polymer applications, particularly with regards to waste valorisation and circular bioeconomy strategies. Legislation and policies that set standards and provide incentives are expected to further boost market demand and R&D.

## Conflicts of interest

The authors declare no conflicts of interest.

## Author contributions

SKR wrote the paper and analysed the data; BS wrote the paper and financed the project; MK wrote the paper and prepared pictures; IK wrote the paper; OP wrote the paper and co‐edited the manuscript; AV wrote the paper and co‐edited the manuscript; PJ wrote the paper; BL supervised and financed the project; UN conceived, designed and financed the project and wrote the paper.

## References

[1] Senila L , Kovacs E , Resz MA , Senila M , Becze A and Roman C (2024) Life cycle assessment (LCA) of bioplastics production from Lignocellulosic waste (study case: PLA and PHB). Polymers 16, 3330. 39684075 10.3390/polym16233330 PMC11644457

[2] Abbate E , Garmendia Aguirre I , Bracalente I , Mancini G and Bennett D (2024) Safe and Sustainable by Design Chemicals and Materials –Methodological Guidance. Publications Office of the European Union, Luxembourg.

[3] Gundlapalli M and Ganesan S (2025) Polyhydroxyalkanoates (PHAs): key challenges in production and sustainable strategies for cost reduction within a circular economy framework. Results Eng 26, 105345.

[4] Negrete‐Bolagay D and Guerrero VH (2024) Opportunities and challenges in the application of bioplastics: perspectives from formulation, processing, and performance. Polymers 16, 2561. 39339026 10.3390/polym16182561 PMC11434805

[5] Vert M , Doi Y , Hellwich KH , Hess M , Hodge P , Kubisa P , Rinaudo M and Schué F (2012) Terminology for biorelated polymers and applications (IUPAC recommendations 2012). Pure Appl Chem 84, 377–410.

[6] European Bioplastics e.V What are bioplastics? https://www.european‐bioplastics.org/bioplastics/

[7] Rosenboom JG , Langer R and Traverso G (2022) Bioplastics for a circular economy. Nat Rev Mater 7, 117–137. 35075395 10.1038/s41578-021-00407-8 PMC8771173

[8] Eriksson FA , Krång A‐S , Michela M , Arato E , Voronova V , Soborowski R , Stagnaro P and Barbir J (2024) Bio‐plastics europe policy brief: bio‐based and biodegradable materials. https://bioplasticseurope.eu/media/pages/policy‐framework/5be43f5eda‐1708592622/d5.4_policy‐brief_bioplastics‐europe_v.1.0.pdf

[9] European Commission (2022) EU policy framework on biobased, biodegradable and compostable plastics. COM(2022) 682 final.

[10] Molina‐Besch K (2022) Use phase and end‐of‐life modeling of biobased biodegradable plastics in life cycle assessment: a review. Clean Technol Environ Policy 24, 3253–3272.

[11] Wolf M , Chomkhamsri K , Brandao M , Pant R , Ardente F , Pennington D , Manfredi S , De Camillis C and Goralczyk M (2010) International Reference Life Cycle Data System (ILCD) Handbook ‐ General Guide for Life Cycle Assessment – Detailed Guidance. Publications Office of the European Union, Luxembourg.

[12] ISO (2006) ISO 14040:2006 Environmental management — Life cycle assessment — Principles and framework.

[13] Ita‐Nagy D , Vázquez‐Rowe I , Kahhat R , Chinga‐Carrasco G and Quispe I (2020) Reviewing environmental life cycle impacts of biobased polymers: current trends and methodological challenges. Int J LCA 25, 2169–2189.

[14] ISO (2006) SO 14044:2006 Environmental management — Life cycle assessment — Requirements and guidelines.

[15] Fantke P , Bijster M , Guignard C , Hauschild M , Huijbregts M , Jolliet O , Kounina A , Magaud V , Margni M , McKon T *et al*. (2017) The USEtox Model. https://usetox.org/model

[16] Caldeira C , Farcal R , Garmendia Aquirre I , Mancini L , Tosches D , Amelio A , Rasmussen K , Rauscher H , Riego SIntes J and Sala S (2022) Safe and Sustainable by Design Chemicals and Materials. Publication Office of the European Union, Luxembourg.

[17] European Commision (2022) Strategic Research and Innovation Plan for Safe and Sustainable Chemicals and Materials Research and Innovation. Publication Office of the European Union, Luxembourg.

[18] Kostapanou A , Chatzipanagiotou KR , Damilos S , Petrakli F and Koumoulos EP (2024) Safe‐and‐sustainable‐by‐design framework: (Re‐)designing the advanced materials lifecycle. Sustainability 16, 10439.

[19] European Commision (2022) COMMISSION RECOMMENDATION (EU) 2022/2510 of 8 December 2022 establishing a European assessment framework for ‘safe and sustainable by design’ chemicals and materials. Off J Eur Union 1, 1–27.

[20] Apel C , Kümmerer K , Sudheshwar A , Nowack B , Som C , Colin C , Walter L , Breukelaar J , Meeus M , Ildefonso B *et al*. (2024) Safe‐and‐sustainable‐by‐design: state of the art approaches and lessons learned from value chain perspectives. Curr Opin Green Sustain Chem 45, 100876.

[21] OECD (2024) Building Trist and Engancing DIalogue for Safe‐andSustainable‐by‐Design (SSbD) Innovation: Developing Tools to Enhace Trusted Environments. OECD Publishing, Paris.

[22] Jayakumar A , Radoor S , Siengchin S , Shin GH and Kim JT (2023) Recent progress of bioplastics in their properties, standards, certifications and regulations: a review. Sci Total Environ 878, 163156. 37003328 10.1016/j.scitotenv.2023.163156

[23] Pooja N , Chakraborty I , Rahman MH and Mazumder N (2023) An insight on sources and biodegradation of bioplastics: a review. 3 Biotech 13, 220. 10.1007/s13205-023-03638-4 PMC10230146 37265543

[24] Agarwal A , Shaida B , Rastogi M and Singh NB (2023) Food packaging materials with special reference to biopolymers‐properties and applications. Chemistry Africa 6, 117–144.

[25] Perera KY , Jaiswal AK and Jaiswal S (2023) Biopolymer‐based sustainable food packaging materials: challenges, solutions, and applications. Foods 12, 2422. 37372632 10.3390/foods12122422 PMC10297947

[26] Flórez M , Cazón P and Vázquez M (2023) Selected biopolymers' processing and their applications: a review. Polymers 15, 641. 36771942 10.3390/polym15030641 PMC9919854

[27] Vasile C and Baican M (2023) Lignins as promising renewable biopolymers and bioactive compounds for high‐performance materials. Polymers 15, 3177. 37571069 10.3390/polym15153177 PMC10420922

[28] Ebhodaghe Samuel Ogbeideand NH (2022) Mechanical properties of biopolymers. In Handbook of Biopolymers ( Thomas Sabuand Ar A , ed.), pp. 1–16. Springer Nature Singapore, Singapore.

[29] Boey JY , Lee CK and Tay GS (2022) Factors affecting mechanical properties of reinforced bioplastics: a review. Polymers 14, 3737. 36145883 10.3390/polym14183737 PMC9505779

[30] Turco R , Santagata G , Corrado I , Pezzella C and Di Serio M (2021) *In vivo* and post‐synthesis strategies to enhance the properties of PHB‐based materials: a review. Front Bioeng Biotechnol 8, 619266. 33585417 10.3389/fbioe.2020.619266 PMC7874203

[31] Ibrahim MI , Alsafadi D , Alamry KA and Hussein MA (2021) Properties and applications of poly(3‐hydroxybutyrate‐co‐3‐hydroxyvalerate) biocomposites. J Polym Environ 29, 1010–1030.

[32] Silva FAGS , Dourado F , Gama M and Poças F (2020) Nanocellulose bio‐based composites for food packaging. Nanomaterials 10, 2041. 33081126 10.3390/nano10102041 PMC7602726

[33] Wang Z , Xu C , Qi L and Chen C (2024) Chemical modification of polysaccharides for sustainable bioplastics. Trends Chem 6, 314–331.

[34] Zhang X , Yin M , Wang J , Pang C , Liu X and Zhu J (2024) Biobased high barrier copolyesters derived from furandicarboxylic acid and citric acid. Eur Polym J 213, 113075.

[35] Lavrič G , Oberlintner A , Filipova I , Novak U , Likozar B and Vrabič‐Brodnjak U (2021) Functional nanocellulose, alginate and chitosan nanocomposites designed as active film packaging materials. Polymers 13, 2523. 34372125 10.3390/polym13152523 PMC8348297

[36] Yilmaz Atay H (2019) Antibacterial activity of chitosan‐based systems. In Functional Chitosan: Drug Delivery and Biomedical Applications ( Sougataand J , ed.), pp. 457–489. Springer Singapore, Singapore.

[37] Jabalera Y , Dominguez‐Gasca N , Muñoz A , Hincke M , Jimenez‐Lopez C and Rodriguez‐Navarro AB (2022) Antimicrobial defenses of table eggs: importance of antibacterial proteins in egg white as a function of hen age in an extended production cycle. Food Microbiol 107, 104068. 35953175 10.1016/j.fm.2022.104068

[38] Fredi G and Dorigato A (2024) Compatibilization of biopolymer blends: a review. Adv Ind Eng Poly Res 7, 373–404.

[39] Mäder G , Rüegg N , Tschichold T and Yildirim S (2025) Utilizing spent coffee grounds as sustainable fillers in biopolymer composites: influence of particle size and content. Sustain Food Technol 3, 1151–1163.

[40] Hernández‐López G , Barrera‐Necha LL , Bautista‐Baños S , Hernández‐López M , Pérez‐Camacho O , Benítez‐Jiménez JJ , Acosta‐Rodríguez JL and Correa‐Pacheco ZN (2025) Characterization of coffee waste‐based biopolymer composite blends for packaging development. Foods 14, 1991. 40509519 10.3390/foods14111991 PMC12155167

[41] Dhal MK , Madhu K , Banerjee A , Prasannavenkadesan V , Kumar A and Katiyar V (2023) Polylactic acid/polycaprolactone/sawdust based biocomposites trays with enhanced compostability. Int J Biol Macromol 253, 126977. 37739287 10.1016/j.ijbiomac.2023.126977

[42] Delgado‐Orti C , Navas‐Martos FJ , Rodríguez‐Liébana JA , Rubia MDL and Jurado‐Contreras S (2024) Development of PLA–waste paper biocomposites with high cellulose content. Polymers 16, 2000. 39065317 10.3390/polym16142000 PMC11280784

[43] Kumar V , Sehgal R and Gupta R (2021) Blends and composites of polyhydroxyalkanoates (PHAs) and their applications. Eur Polym J 161, 110824.

[44] Bolka S , Pešl T , Rebeka L , Rozman T , Bobovnik R and Nardin B (2021) Toughness modification of PLA based blends with nanocrystalline cellulose.

[45] Carrasco F , Pérez OS , Albiter NL and Maspoch ML (2023) Improvement of the thermal stability of polymer bioblends by means of reactive extrusion. Polymers 15, 105. 10.3390/polym15010105 PMC9824162 36616455

[46] Ozah R and Sarma D (2025) Performance optimization of biopolymer in material extrusion process using support vector regression and Grey Wolf algorithm. J Mater Eng Perform 34, 24600–24610.

[47] Nešić A , Lorber R , Bolka S , Nardin B and Pilić B (2025) Additive‐free multiple processing of PLA pre‐consumer waste: influence on mechanical and thermal properties. Polymers 17, 2164. 40871112 10.3390/polym17162164 PMC12388883

[48] European Bioplastics (2024) Bioplastic Market Development Update 2024. European Bioplastics, Germany. https://docs.european‐bioplastics.org/publications/market_data/2024/EUBP_Market_Data_Report_2024.pdf

[49] European Bioplastics (2023) Bioplastics Market Development Update 2023. European Bioplastics, Germany. https://docs.european‐bioplastics.org/publications/market_data/2023/EUBP_Market_Data_Report_2023.pdf

[50] Das A , Ringu T , Ghosh S and Pramanik N (2023) A comprehensive review on recent advances in preparation, physicochemical characterization, and bioengineering applications of biopolymers. Polymer Bulletin 80, 7247–7312. 36043186 10.1007/s00289-022-04443-4 PMC9409625

[51] Borah AR , Hazarika P , Duarah R , Goswami R and Hazarika S (2024) Biodegradable electrospun membranes for sustainable industrial applications. ACS Omega 9, 11129–11147. 38496999 10.1021/acsomega.3c09564 PMC10938411

[52] Šmid S , Verbič A , Zemljič LF and Gorjanc M (2023) Eco‐finishing of cotton with chitosan and Giant goldenrod (Solidago gigantea Aiton) aqueous extract for development of antioxidant and UV protective textiles. J Nat Fibers 20, 2253371.

[53] Pretschuh C , Mihalic M , Sponner C , Lummerstorfer T , Steurer A and Unterweger C (2025) Physical foam injection molding of cellulose fiber reinforced polypropylene by using CO_2_: parameter variation and comparison to chemical foam injection molding. J Compos Sci 9, 50.

[54] Shigrekar M and Amdoskar V (2024) A review on recent progress and techniques used for fabricating superhydrophobic coatings derived from biobased materials. RSC Adv 14, 32668–32699. 39421684 10.1039/d4ra04767b PMC11483902

[55] Campanale C , Galafassi S , Di Pippo F , Pojar I , Massarelli C and Uricchio VF (2024) A critical review of biodegradable plastic mulch films in agriculture: definitions, scientific background and potential impacts. TrAC 170, 117391.

[56] Farias AR , Domingos S , Possidónio C , Cruz B and Luís S (2024) REPORT ON DELIVERABLE 6.1 – Risk management strategies & market opportunities for commercialisation of new biopolymer production technologies.

[57] Vicente FA , Hren R , Novak U , Čuček L , Likozar B and Vujanović A (2024) Energy demand distribution and environmental impact assessment of chitosan production from shrimp shells. Renew Sustain Energy Rev 192, 114204.

[58] Zimmermann L , Dombrowski A , Völker C and Wagner M (2020) Are bioplastics and plant‐based materials safer than conventional plastics? *in vitro* toxicity and chemical composition. Environ Int 145, 106066. 32951901 10.1016/j.envint.2020.106066

[59] Lestido‐Cardama A , Barbosa‐Pereira L , Sendón R , Bustos J , Paseiro Losada P and de Rodríguez Bernaldo Quirós A (2025) Chemical safety and risk assessment of bio‐based and/or biodegradable polymers for food contact: a review. Food Res Int 202, 115737. 39967183 10.1016/j.foodres.2025.115737

[60] Nova Mechanics & Entelos Institute (2025) chemPharos: Developed by Scientists, Curated for Excellence Curated Datasets ready for Modelling. https://db.chempharos.eu/datasets/Datasets.zul?datasetID=ds13

[61] Liu J , Xia P , Qu Y , Zhang X , Shen R , Yang P , Tan H , Chen H and Deng Y (2025) Long‐term exposure to environmentally realistic doses of starch‐based microplastics suggests widespread health effects. J Agric Food Chem 73, 9867–9878. 40202198 10.1021/acs.jafc.4c10855

[62] Shi W , Fuad ARM , Li Y , Wang Y , Huang J , Du R , Wang G , Wang Y and Yin T (2023) Biodegradable polymeric nanoparticles increase risk of cardiovascular diseases by inducing endothelium dysfunction and inflammation. J Nanobiotechnol 21, 65. 10.1186/s12951-023-01808-3 PMC9951517 36829180

[63] Yanamala N , Farcas MT , Hatfield MK , Kisin ER , Kagan VE , Geraci CL and Shvedova AA (2014) *In vivo* evaluation of the pulmonary toxicity of cellulose nanocrystals: a renewable and sustainable nanomaterial of the future. ACS Sustain Chem Eng 2, 1691–1698. 26753107 10.1021/sc500153k PMC4703331

[64] Yamashita Y , Tokunaga A , Aoki K , Ishizuka T , Fujita S and Tanoue S (2025) Safety of mechanically fibrillated cellulose nanofibers (CNFs) by inhalation exposure based on TG412. Nanomaterials 15, 214. 39940189 10.3390/nano15030214 PMC11819688

[65] Sai S , Mani R , Vijayakumar P , Ganesan M , Velu K , Ayyamperumal R , Rajagopal R , Chang SW , Alfarhan A and Ravindran B (2022) Risk assessment of potential toxicity induced by bio and synthetic plastic microspheres in Lates calcarifer. Chemosphere 298, 134269. 35307385 10.1016/j.chemosphere.2022.134269

[66] Abe MM , Branciforti MC , Nallin Montagnolli R , Marin Morales MA , Jacobus AP and Brienzo M (2022) Production and assessment of the biodegradation and ecotoxicity of xylan‐ and starch‐based bioplastics. Chemosphere 287, 132290. 34562707 10.1016/j.chemosphere.2021.132290

[67] Bernardi AD , Bandini F , Marini E , Tagliabue F , Casucci C , Brunetti G , Vaccari F , Bellotti G , Tabaglio V , Fiorini A *et al*. (2024) Integrated assessment of the chemical, microbiological and ecotoxicological effects of a bio‐packaging end‐of‐life in compost. Sci Total Environ 951, 175403. 39128510 10.1016/j.scitotenv.2024.175403

[68] Boisseaux P , Hopkinson P , Santillo D , Smith C , Garmulewicz A , Powell Z and Galloway T (2023) Environmental safety of second and third generation bioplastics in the context of the circular economy. Ecotoxicol Environ Saf 256, 114835. 37003058 10.1016/j.ecoenv.2023.114835

[69] Koumentakou I , Meretoudi A , Emmanouil C and Kyzas GZ (2024) Environmental toxicity and biodegradation of chitosan derivatives: a comprehensive review. J Ind Eng Chem 146, 70–86.

[70] Venâncio C , Lopes I and Oliveira M (2022) Bioplastics: known effects and potential consequences to marine and estuarine ecosystem services. Chemosphere 309, 136810. 36228730 10.1016/j.chemosphere.2022.136810

[71] Cucina M (2023) The lesser of two evils: enhancing biodegradable bioplastics use to fight plastic pollution requires policy makers interventions in Europe. Environmental Impact Assessment Review 103, 107230.

[72] Edo GI , Mafe AN , Ali ABM , Akpoghelie PO , Yousif E , Isoje EF , Igbuku UA , Zainulabdeen K , Owheruo JO , Essaghah AEA *et al*. (2025) Life cycle and environmental impact assessment of biopolymer‐based packaging vs. conventional plastics in the food industry. Mater Today Commun 46, 112806.

[73] Kogje M , Satdive A , Mestry S and Mhaske ST (2025) Biopolymers: a comprehensive review of sustainability, environmental impact, and lifecycle analysis. Iran Polym J 34, 1481–1524.

[74] Roy D , Dey AK , Mandal A and Kamila B (2025) A comprehensive review on the sustainable approach of fossil‐based polymers toward bio‐based polymers. Polymer Bulletin 82, 11625–11696.

[75] Zuiderveen EAR , Kuipers KJJ , Caldeira C , Hanssen SV , van der Hulst MK , de Jonge MMJ , Vlysidis A , van Zelm R , Sala S and Huijbregts MAJ (2023) The potential of emerging bio‐based products to reduce environmental impacts. Nat Commun 14, 8521. 38129383 10.1038/s41467-023-43797-9 PMC10739733

[76] Yadav K and Nikalje GC (2024) Comprehensive analysis of bioplastics: life cycle assessment, waste management, biodiversity impact, and sustainable mitigation strategies. PeerJ 12, e18013. 39282116 10.7717/peerj.18013 PMC11401513

[77] Ali SS , Abdelkarim EA , Elsamahy T , Al‐Tohamy R , Li F , Kornaros M , Zuorro A , Zhu D and Sun J (2023) Bioplastic production in terms of life cycle assessment: a state‐of‐the‐art review. Environ Sci Ecotech 15, 100254. 10.1016/j.ese.2023.100254 PMC10068114 37020495

[78] Kuroda H , Amasawa E , Nakatani J and Hirao M (2023) Linear programming approach to design bio‐based plastics strategies for Japan: integration of material characteristics, product applications, and end of life options. Resour Conserv Recycl 198, 107137.

[79] Pereira BAM , Martins NO , Dantas SC and Lima AM (2025) Evaluation of the substitution of polyethylene for polylactic acid in sanitary pads through life cycle assessment. Sustain Sci Tech 2, e024002.

[80] Synani K , Abeliotis K , Velonia K , Maragkaki A , Manios T and Lasaridi K (2024) Environmental impact and sustainability of bioplastic production from food waste. Sustainability 16, 5529.

[81] Rosa ADL , Francois J‐M and Auriol C (2025) Ex‐ante LCA of a novel biodegradable polymer based on 2,4‐dihydroxybutyric acid produced from renewable carbon feedstock. IFAC‐PapersOnLine 59, 2463–2467.

[82] Luo C , Zhou Y , Chen Z , Bian X , Chen N , Li J , Wu Y and Yang Z (2024) Comparative life cycle assessment of PBAT from fossil‐based and second‐generation generation bio‐based feedstocks. Sci Total Environ 954, 176421. 39306119 10.1016/j.scitotenv.2024.176421

[83] Lee S , Lee I , Seo D , Kim H , Joo G , Lee S and Park K (2024) Life cycle assessment of aPHA production. ACS Sustain Chem Eng 12, 72–84.

[84] Ritzen L , Sprecher B , Bakker C and Balkenende R (2024) Sustainability of bio‐based polyethylene: the influence of biomass sourcing and end‐of‐life. J Ind Ecol 28, 1684–1698.

[85] Chalermthai B , Nootong K , Olsen BD , Assabumrungrat S and Charoensuppanimit P (2024) Cradle‐to‐gate life cycle assessment of spirulina bioplastic produced via plasticization with glycerol. Environ Res 251, 118622. 38442816 10.1016/j.envres.2024.118622

[86] Newby DJ , El‐Ajouz MA , Mai AM and Hobbs SR (2025) Life cycle assessment of polylactic acid municipal waste disposal in Belize. Environ Res Lett 20, e084076.

[87] Ghannadzadeh A and van der Meer Y (2025) Combining dynamic life cycle assessment and net ecosystem exchange through the framework of biobased materials and products‐life cycle assessment (BBM‐LCA): application to polylactic acid. Biofuels Bioprod Biorefin 19, 1075–1087.

[88] Li J , Wang Y , Xu C , Liu S , Dai J and Lan K (2024) Bioplastic derived from corn stover: life cycle assessment and artificial intelligence‐based analysis of uncertainty and variability. Sci Total Environ 946, 174349. 38944302 10.1016/j.scitotenv.2024.174349

[89] Nhu TT , Boone L , Guillard V , Chatellard L , Reis M , Matos M and Dewulf J (2024) Environmental sustainability assessment of biodegradable bio‐based poly(3‐hydroxybutyrate‐co‐3‐hydroxyvalerate) from agro‐residues: production and end‐of‐life scenarios. J Environ Manage 356, 120522. 38493645 10.1016/j.jenvman.2024.120522

[90] Schwarz A , Ferjan Š and Kunst J (2023) Life cycle assessment of advanced grade PLA product with novel end‐of‐life treatment through depolymerization. Sci Total Environ 905, 167020. 37714343 10.1016/j.scitotenv.2023.167020

[91] Simon Suwanzy Dzreke and Semefa Elikplim Dzreke (2025) Life cycle assessment (LCA) and supply chain network optimization for sustainable integration of bio‐based polymers (PLA/PHA) in regional packaging systems. Eng Sci Technol J 6, 355–374.

[92] Ayala M , Goosen N , Michalak L , Thomsen M and Pizzol M (2024) Prospective LCA of brown seaweed‐based bioplastic: upscaling from pilot to industrial scale. Sustainable Prod Consumption 52, 416–426.

[93] de Souza NRD , Matt L , Sedrik R , Vares L and Cherubini F (2023) Integrating ex‐ante and prospective life‐cycle assessment for advancing the environmental impact analysis of emerging bio‐based technologies. Sustainable Prod Consumption 43, 319–332.

[94] Keyes A , Saffron CM , Manjure S and Narayan R (2024) Biobased compostable plastics end‐of‐life: environmental assessment including carbon footprint and microplastic impacts. Polymers 16, 3073. 39518282 10.3390/polym16213073 PMC11548190

[95] Rajeshkumar G , Arvindh Seshadri S , Devnani GL , Sanjay MR , Siengchin S , Prakash Maran J , Al‐Dhabi NA , Karuppiah P , Mariadhas VA , Sivarajasekar N *et al*. (2021) Environment friendly, renewable and sustainable poly lactic acid (PLA) based natural fiber reinforced composites – a comprehensive review. J Clean Prod 310, 127483.

[96] Roy Chong JW , Tan X , Khoo KS , Ng HS , Jonglertjunya W , Yew GY and Show PL (2022) Microalgae‐based bioplastics: future solution towards mitigation of plastic wastes. Environ Res 206, 112620. 34968431 10.1016/j.envres.2021.112620

[97] Gurunathan MK , Navasingh RJH , Selvam JDR and Čep R (2025) Development and characterization of starch bioplastics as a sustainable alternative for packaging. Sci Rep 15, 15264. 40312412 10.1038/s41598-025-00221-0 PMC12046032

[98] Islam T , Chaion MH , Jalil MA , Rafi AS , Mushtari F , Dhar AK and Hossain S (2024) Advancements and challenges in natural fiber‐reinforced hybrid composites: a comprehensive review. SPE Polym 5, 481–506.

[99] Caldeira C , Abbate E , Moretti C , Mancini L and Sala S (2024) Safe and sustainable chemicals and materials: a review of sustainability assessment frameworks. Green Chem 26, 7456–7477.

[100] Elser I and Buchmeiser MR (2024) Toward sustainable fiber‐reinforced polymer composites. Macromol Mater Eng 309, 2400013.

[101] Marques, F. R. , Maryoret, M. , Cabrera Gonzalez, V. , Harasek, M. , Ramonet, F. & Cabrera‐Gonzalez, M. (2025) Promoting Safe Operations in Biorefineries: A Comprehensive Safe and Sustainable‐by‐Design Framework. Proceedings of 33rd European Biomass Conference and Exhibition. Valencia, Spain. ETA Florence Renewable Energies, Florence.

[102] Loh YZ and Chew IML (2021) Mitigating plastic pollution through better process design: an opportunity from biomass to bioplastic. Biomass Conv Bioref 15, 29781–29802.

[103] Todea A , Bîtcan I , Giannetto M , Rădoi II , Bruschi R , Renzi M , Anselmi S , Provenza F , Bentivoglio T , Asaro F *et al*. (2024) Enzymatic synthesis and structural modeling of bio‐based Oligoesters as an approach for the fast screening of marine biodegradation and Ecotoxicity. Int J Mol Sci 25, 5433. 38791471 10.3390/ijms25105433 PMC11121971

[104] Oliaei E , Josephson P , Montanari C , Berglund LA and Olsén P (2025) Fully biobased circular biocomposites for chemical recycling to monomer and fiber. Compos Part B Eng 306, 112814.

[105] Zappaterra F , Todea A , Asaro F , Ditalia PFA , Danielli C , Renzi M , Anselmi S and Gardossi L (2025) Integrating computational and experimental methods for the rational Ecodesign and synthesis of functionalized safe and sustainable biobased Oligoesters. Polymers 17, 2537. 41012299 10.3390/polym17182537 PMC12473331

[106] Rubalcaba Medina A , Hansen SF , Rodriguez Macias FJ and Baun A (2024) A design‐phase environmental safe‐and‐sustainable‐by‐design categorization tool for the development and innovation of nano‐enabled advanced materials (AdMaCat). Environ Sci Nano 11, 3761–3773.

[107] Smith HA , Cabling LPB , Leonard NA , Dubrawski KL and Buckley HL (2024) Green alginate extraction from Macrocystis pyrifera for bioplastic applications: physicochemical, environmental impact, and chemical Hazard analyses. ACS Sustainable Resour Manage 1, 958–969.

[108] Bhat MA , Radu T , Martín‐Fabiani I , Kolokathis PD , Papadiamantis AG , Wagner S , Kohl Y , Witters H , Gebbink WA , Rodriguez YP *et al*. (2025) Safe and sustainable by design of next generation chemicals and materials: SSbD4CheM project innovations in the textiles, cosmetic and automotive sectors. Comput Struct Biotechnol J 29, 60–71. 40212339 10.1016/j.csbj.2025.03.022 PMC11984538

[109] Deliane F , Amon M‐C , Dulucq C , Sevrin C , Direur G , Mezy A and Schmitt C (2025) Development of PFAS‐free sol gel coating in textile applications based on a Safe and Sustainable by Design (SSbD) strategy. Proceedings of European Coatings Show Conference 2025. Vincentz Network, Nuremberg, Germany.

[110] Yu Y , Astner AF , Zahid TM , Chowdhury I , Hayes DG and Flury M (2023) Aggregation kinetics and stability of biodegradable nanoplastics in aquatic environments: effects of UV‐weathering and proteins. Water Res 239, 120018. 37201372 10.1016/j.watres.2023.120018

[111] Ali W , Ali H , Gillani S , Zinck P and Souissi S (2023) Polylactic acid synthesis, biodegradability, conversion to microplastics and toxicity: a review. Environ Chem Lett 21, 1761–1786.

[112] Poli V , Lavagnolo MC , Basaglia M , Bonato T , Zanatta S and Modesti M (2025) Assessment of the biodegradability of polylactic acid (PLA) in freshwater using EN ISO 14851:2019: challenges and outcomes. J Hazard Mater 491, 137974. 40117770 10.1016/j.jhazmat.2025.137974

[113] Mizuno W , Sano M , Song C , Nakatani T and Takeuchi S (2003) Evaluation of biodegradability of several biodegradable plastics in natural environments. Kobunshi Ronbunshu 60, 622–628.

[114] Nabeoka R , Suzuki H , Akasaka Y , Ando N and Yoshida T (2021) Evaluating the ready biodegradability of biodegradable plastics. Environ Toxicol Chem 40, 2443–2449. 34003509 10.1002/etc.5116

[115] Cao Z , Kim C , Li Z and Jung J (2024) Comparing environmental fate and ecotoxicity of conventional and biodegradable plastics: a critical review. Sci Total Environ 951, 175735. 39187074 10.1016/j.scitotenv.2024.175735

[116] Pires JRA , Souza VGL , Fuciños P , Pastrana L and Fernando AL (2022) Methodologies to assess the biodegradability of bio‐based polymers—current knowledge and existing gaps. Polymers 14, 1359. 35406232 10.3390/polym14071359 PMC9002992

[117] Lavagnolo MC , Poli V , Zampini AM and Grossule V (2024) Biodegradability of bioplastics in different aquatic environments: a systematic review. J Environ Sci (China) 142, 169–181. 38527882 10.1016/j.jes.2023.06.013

[118] Ali S and Chang Y‐C (2023) Ecotoxicological impact of bioplastics biodegradation: a comprehensive review. Processes 11, 3445.

[119] Lee ES , Park SY and Kim CG (2024) Comparison of anaerobic digestion of starch‐ and petro‐based bioplastic under hydrogen‐rich conditions. Waste Manag 175, 133–145. 38194798 10.1016/j.wasman.2023.12.050

[120] Vardar S , Demirel B and Onay TT (2022) Degradability of bioplastics in anaerobic digestion systems and their effects on biogas production: a review. Rev Environ Sci Biotechnol 21, 205–223.

[121] Benn N and Zitomer D (2018) Pretreatment and anaerobic Co‐digestion of selected PHB and PLA bioplastics. Front Environ Sci 5, 93.

[122] Jiang X , Bi D , Cheng Y , Wang S , Peng BY , Shen H , Zhang T , Xia X , Shen Z and Zhang Y (2023) Enzyme pretreatments for anaerobic co‐digestion of food waste blended with bioplastics: effects on methane production and microbial community structure. New J Chem 47, 20846–20858.

[123] Cazaudehore G , Guyoneaud R , Evon P , Martin‐Closas L , Pelacho AM , Raynaud C and Monlau F (2022) Can anaerobic digestion be a suitable end‐of‐life scenario for biodegradable plastics? A critical review of the current situation, hurdles, and challenges. Biotechnol Adv 56, 107916. 35122986 10.1016/j.biotechadv.2022.107916

[124] Quecholac‐Piña X , Hernández‐Berriel M d C , Mañón‐Salas M d C , Espinosa‐Valdemar RM and Vázquez‐Morillas A (2020) Degradation of plastics under anaerobic conditions: a short review. Polymers 12, 109. 31948016 10.3390/polym12010109 PMC7023122

[125] Zhang Y , Wang Z , Wang F , Zhou H , Zhang L and Xie B (2024) Anaerobic degradation of aromatic and aliphatic biodegradable plastics: potential mechanisms and pathways. Environ Sci Technol 58, 19462–19474. 39424349 10.1021/acs.est.4c07554

[126] Falzarano M , Polettini A , Pomi R , Rossi A and Zonfa T (2023) Anaerobic biodegradability of commercial bioplastic products: systematic bibliographic analysis and critical assessment of the latest advances. Materials 16, 2216. 36984096 10.3390/ma16062216 PMC10058929

[127] Jin Y , Cai F , Song C , Liu G and Chen C (2022) Degradation of biodegradable plastics by anaerobic digestion: morphological, micro‐structural changes and microbial community dynamics. Sci Total Environ 834, 155167. 35421475 10.1016/j.scitotenv.2022.155167

[128] Derkenne P , Chatellard L , Béline F , Pierson‐Wickmann AC , Gontard N and Dabert P (2025) Understanding the biodegradation of PHBV/cellulose composites in mesophilic anaerobic digestion. Sci Total Environ 959, 178224.

[129] García‐Depraect O , Lebrero R , Rodriguez‐Vega S , Bordel S , Santos‐Beneit F , Martínez‐Mendoza LJ , Aragão Börner R , Börner T and Muñoz R (2022) Biodegradation of bioplastics under aerobic and anaerobic aqueous conditions: kinetics, carbon fate and particle size effect. Bioresour Technol 344, 126265. 34737051 10.1016/j.biortech.2021.126265

[130] Zhao L , Wang P , Li Y , Yu M , Zheng Y , Ren L , Wang Y and Li J (2024) Feasibility of anaerobic co‐digestion of biodegradable plastics with food waste, investigation of microbial diversity and digestate phytotoxicity. Bioresour Technol 393, 130029. 37977495 10.1016/j.biortech.2023.130029

[131] Emadian SM , Onay TT and Demirel B (2017) Biodegradation of bioplastics in natural environments. Waste Manag 59, 526–536. 27742230 10.1016/j.wasman.2016.10.006

[132] Samir A , Ashour FH , Hakim AAA and Bassyouni M (2022) Recent advances in biodegradable polymers for sustainable applications. NPJ Mater Degrad 6, 68.

[133] Akinsemolu AA , Idowu AM and Onyeaka HN (2024) Recycling Technologies for Biopolymers: current challenges and future directions. Polymers 16, 2770. 39408479 10.3390/polym16192770 PMC11478719

[134] Gioia C , Giacobazzi G , Vannini M , Totaro G , Sisti L , Colonna M , Marchese P and Celli A (2021) End of life of biodegradable plastics: composting versus Re/upcycling. ChemSusChem 14, 4167–4175. 34363734 10.1002/cssc.202101226 PMC8518687

[135] Mudhoo A , Mohee R , Unmar GD and Sharma SK (2011) Degradation of biodegradable and green polymers in the composting environment. In A Handbook of Applied Biopolymer Technology: Synthesis, Degradation and Applications ( Sharma SK and Mudhoo A , eds), The Royal Society of Chemistry, Cambridge.

[136] Kalita NK , Hazarika D , Kalamdhad A and Katiyar V (2021) Biodegradation of biopolymeric composites and blends under different environmental conditions: approach towards end‐of‐life panacea for crop sustainability. Bioresour Technol Rep 15, 100705.

[137] Afshar SV , Boldrin A , Astrup TF , Daugaard AE and Hartmann NB (2024) Degradation of biodegradable plastics in waste management systems and the open environment: a critical review. J Clean Prod 434, 140000.

[138] D'Amario J , Limsukon W , Bher A and Auras R (2025) Impact of hydrolysis pretreatment on the compostability of biodegradable poly(caprolactone) and poly(lactic acid) films. RSC Applied Polymers 3, 711–721.

[139] Lors C , Leleux P and Park CH (2024) State of the art on biodegradability of bio‐based plastics containing polylactic acid. Front Mater 11, 1476484.

[140] Kunioka M , Ninomiya F and Funabashi M (2009) Biodegradation of poly(butylene succinate) powder in a controlled compost at 58 °C evaluated by naturally‐occurring carbon 14 amounts in evolved CO_2_ based on the ISO 14855‐2 method. Int J Mol Sci 10, 4267–4283. 20057944 10.3390/ijms10104267 PMC2790107

[141] Narmon AS , van Slagmaat CAMR , De Wildeman SMA and Dusselier M (2023) Sustainable Polythioesters via Thio(no)lactones: monomer synthesis, ring‐opening polymerization, end‐of‐life considerations, and industrial perspectives. ChemSusChem 16, e202202276. 36649173 10.1002/cssc.202202276

